# Spatial Epidemiology and Ecological Determinants of Ticks and Tick-Borne Pathogens Co—Circulation in Brijuni National Park, Croatia

**DOI:** 10.3390/ijerph23050617

**Published:** 2026-05-07

**Authors:** Maja Cvek, Emina Pustijanac, Marko Vucelja, Dean Girotto, Josip Margaletić, Linda Bjedov

**Affiliations:** 1 Teaching Institute of Public Health of Istria County, Nazorova 23, 52100 Pula, Croatia; maja.cvek@zzjziz.hr; 2 Faculty of Natural Sciences, Juraj Dobrila University of Pula, Zagrebačka 30, 52100 Pula, Croatia; emina.pustijanac@unipu.hr; 3 Faculty of Forestry and Wood Technology, University of Zagreb, Svetošimunska cesta 23, 10000 Zagreb, Croatia; jmargaletic@sumfak.unizg.hr (J.M.); lbjedov@sumfak.unizg.hr (L.B.); 4 Neurosurgery Clinic, Faculty of Medicine, University of Rijeka, 51000 Rijeka, Croatia; neurokirurgija@kbc-rijeka.hr

**Keywords:** spatial epidemiology, pathogen co-circulation, *Ixodes ricinus*, Tick-borne Diseases (TBDs), island ecosystem, GIS mapping

## Abstract

**Highlights:**

**Public health relevance—How does this work relate to a public health issue?**

- Our study focuses on how tick-borne diseases circulate in popular tourist spots like Brijuni National Park, where the interaction between nature and people is very high.
- We wanted to fill the gap in data regarding where and why these pathogens appear, helping to better understand the real risk for anyone visiting or working in Mediterranean island environments.

**Public health significance—Why is this work of significance to public health?**

- We confirmed that three serious pathogens (*Borrelia*, *Anaplasma*, and *Ehrlichia*) are actively present in the park’s tick population.
- Particularly important is our finding that nearly 30% of ticks carry more than one pathogen, which is a big deal for doctors because a single tick bite can cause a much more complex illness than expected.

**Public health implications—What are the key implications or messages for practitioners, policy makers and/or researchers in public health?**

- We discovered that even well-maintained areas like park gardens can hide ticks because of irrigation, meaning tourists might be at risk even where they feel “safe.”
- These findings give park managers clear directions: they can now focus on specific “hotspots” for maintenance and better warn visitors exactly where to be careful.

**Abstract:**

Tick-borne diseases are a growing public health concern in the Mediterranean. Brijuni National Park (BNP), a unique, highly visited island ecosystem characterized by increased large game host density and diverse Mediterranean habitats, presents an elevated risk for pathogen co-circulation. This study addresses the lack of spatial and epidemiological data to accurately assess human exposure risk in this environment. We performed a detailed geospatial and epidemiological risk mapping of pathogen co-circulation in BNP. A total of 587 hard ticks were collected across 26 georeferenced micro-locations (2020–2022). Ticks were morphologically identified and subsequently screened for six key zoonotic bacterial pathogens using qPCR. The Minimal Infection Rate (MIR) and a Co-infection Rate (CR) were calculated. Geographic Information System (GIS) mapping was utilized to map ecological determinants of risk. *Ixodes ricinus* was the overwhelmingly dominant vector (94.0%), peaking in spring, with activity absent in summer. Recorded diverse tick fauna also included *Hyalomma marginatum* (3%), *Haemaphysalis punctata* (2%), *Ixodes frontalis* (0.8%) and *Rhipicephalus sanguineus* (0.2%). Active circulation of *Borrelia burgdorferi* s.l. (Bbsl), *Anaplasma phagocytophilum*, and *Ehrlichia canis* were confirmed. Bbsl presented the highest MIR (3.05). The Co-infection Rate (CR) was notably high at 29.41%, with triple co-infections (Bbsl, *A. phagocytophilum*, *E. canis*) concentrated in cultivated mosaics and holm oak forests (*Quercus ilex* L.). The highest number of ticks was recovered from ecotone zones, accounting for 50.0% of the total catch, confirming them as high-risk interfaces. The absence of *Rickettsia conorii* may be attributed to the strict control/absence of its primary host (domestic dogs). The presence of the exotic vector *H. marginatum* was also confirmed. The high rate of co-infection and the spatial concentration of risk in specific habitats underscore an elevated and complex public health risk in BNP, closely linked to habitat structure and increased game host density. This research provides an essential geospatial framework for targeted ‘One Health’ management, prioritizing vector control in ecotone zones and dense forest refugia. Urgent surveillance for the exotic *H. marginatum* is warranted to monitor the potential risk of Crimean-Congo Hemorrhagic Fever.

## 1. Introduction

Ticks (Acari: Ixodidae) are obligatory hematophagous ectoparasites that pose a significant public health and veterinary problem across the Mediterranean and Eumediterranean regions [1,2,3]. Tick-borne pathogens (TBPs)—including bacteria, viruses, rickettsiae, and protozoa—cause major vector-borne diseases (e.g., tick-borne encephalitis—TBE, Lyme borreliosis—LB, human granulocytic anaplasmosis—HGA), which are increasingly reported across Europe [4,5,6]. The heightened incidence and expanding geographic range of tick-borne diseases (TBDs) are closely linked to climate change and changes in human behavior [7,8,9,10]. Given the crucial role of animal reservoirs in disease maintenance, a global “One Health” approach is essential, integrating human, animal, and environmental health surveillance [11].

In the Republic of Croatia (RC), the most prevalent disease vectors are *Ixodes ricinus* (principal vector for LB and TBE) and *Rhipicephalus sanguineus* [12,13,14,15]. *I. ricinus* is widely distributed, favoring forested areas [16]. Of critical concern is *Hyalomma marginatum*, associated with the transmission of Crimean-Congo Hemorrhagic Fever (CCHF) [17,18]. Although no autochthonous human CCHF cases have been confirmed in RC (November 2025), serological evidence confirms the silent circulation of the CCHF virus in domestic animals (sheep, horses) in coastal and subcoastal regions [19]. Due to the presence of the vector and confirmed virus circulation, active surveillance is mandated by both national and EU priority disease regulations [20], forming the basis of the Croatian “One Health” (CROOH) project [21]. Also of particular significance is the tick *Haemaphysalis punctata*, whose presence has been recorded in coastal grassy and shrubby habitats of Croatia, where it primarily parasitizes cattle, sheep, and horses, and is associated with the transmission of primarily protozoan parasites in livestock [1,22].

The Brijuni National Park (BNP), located on the western Istrian coast, represents a unique and complex island system for vector-borne disease research. Its historical context, dating back to Robert Koch’s successful malaria eradication in 1901, attests to its vector-borne disease potential [23].

The BNP environment is defined by two critical factors: increased host density (the confined populations of large allochthonous game—fallow deer (*Dama dama* L.), axis deer (*Axis axis* Erx.) and mouflon, acting as principal hosts, concentrating host–tick interactions and resulting in an elevated tick sampling yield, and habitat duality where the dominant holm oak forests (*Quercus ilex* L., EU code 9340 [24,25] provide the shade and humidity essential for *I. ricinus* [26]. Simultaneously, the area is exposed to the expansion of thermophilic species like *H. marginatum* [18].

Furthermore, BNP, a crucial Natura 2000 staging site for migratory birds [27], introduces an additional risk layer. The ornithophilic tick *Ixodes frontalis* acts as a biological bridge [28], potentially importing novel pathogens, including *Borrelia* spp. and *Rickettsia* spp., making the island a zone of elevated pathogen co-circulation risk.

Analysis of tick spatial distribution, sex, and developmental stage is crucial for accurately assessing disease risk and comprehending the ecological dynamics of the tick-host system [29]. While studies exist in Istria [14,30,31,32,33], there is a lack of data on pathogen prevalence within ticks [3] and, crucially, a need for spatial data on vector distribution across Europe [34,35]. This research addresses these gaps by focusing on the insufficiently studied BNP to assess the risk of human exposure, particularly given the intense tourist visitation [36]. We aim to analyze the active co-circulation of six key zoonotic bacterial pathogens—*Borrelia burgdorferi* s.l. (Bbsl), *Borrelia myamotoi*, *Rickettsia conorii*, *Anaplasma phagocytophilum*, *Francisella tularensis*, and *Ehrlichia canis*—chosen for their public health significance [3]. Geographic Information System (GIS) modeling is the optimal tool for integrating these multiple risk factors into a unified model and mapping the ecological determinants that shape disease dynamics [37,38]. Despite existing surveillance, no published study has utilized advanced spatial statistical models to map tick species and associated pathogens within the specific confines of the BNP. This research represents the first detailed geospatial and epidemiological risk mapping of pathogen co-circulation in this unique island ecosystem.

## 2. Materials and Methods

### 2.1. Study Area and Tick Sampling

**Figure 1:**
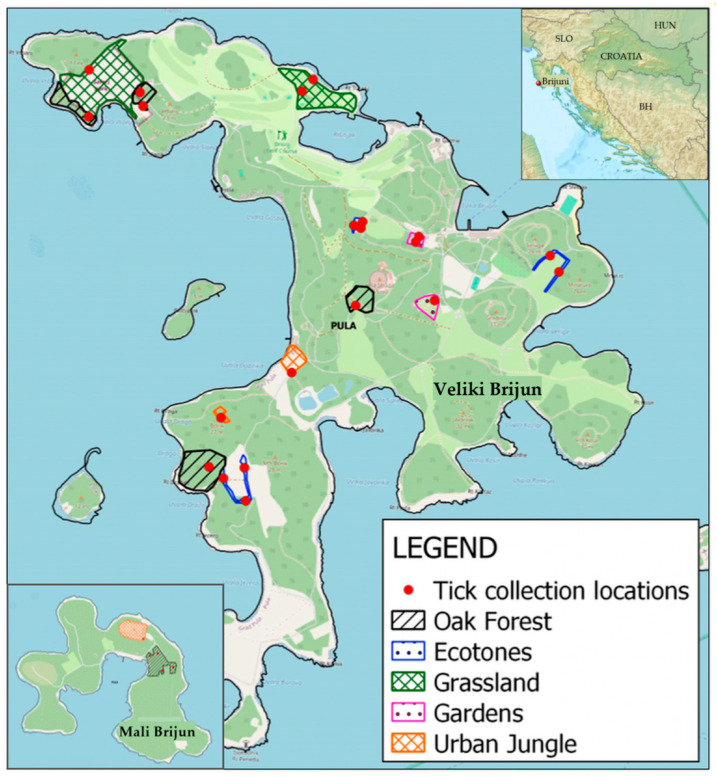
Spatial distribution of tick collection sites i.e., specific habitat types (oak forest, ecotones, grassland, gardens, and urban jungle) in Brijuni National Park (Veliki Brijun and Mali Brijun islands). Note: Due to the close geographical proximity of certain micro-localities, some of the 26 collection points overlap on the map and are represented by a single dot.

The study was conducted in BNP (44°54′ N 13°44′ E) [39], a unique 14-island archipelago selected for its combined ecological factors and elevated epidemiological risk. Tick sampling was performed at 26 georeferenced microlocations on Veliki Brijun and Mali Brijun, two largest islands in the Brijuni archipelago, situated in the northern Adriatic Sea off the southwestern coast of the Istrian peninsula (Figure 1). Sampling was conducted between 15 June 2020, and 30 November 2022. Locations were selected to cover representative habitat types and ecotones—transition zones between ecosystems characterized by high biodiversity.

#### 2.1.1. Habitat Classification and Characterization

Within the framework of ecological research conducted in BNP, habitats were classified based on vegetation structure, microclimatic conditions, and the intensity of anthropogenic and biological pressures. The specific characteristics of this insular ecosystem, marked by high densities of both autochthonous and allochthonous wildlife species (e.g., *D. dama*, *A. axis*), necessitate differentiation among distinct habitat types with varying suitability for tick populations. Habitats were classified based on vegetation structure and microclimatic conditions. The climatogenic holm oak forest (*Fraxino orni—Quercetum ilicis* Horvatić/1956/1958) [24], characterized by a closed canopy and an understory of *Viburnum tinus* and *Ruscus aculeatus*, provides stable thermal and hygrometric refugia within a thick litter layer (humidity 78–85%) essential for *I. ricinus* survival [40,41,42]. Ecotone zones, transition zones dominated by *Pistacia* spp. and *Myrtus communis*, retain soil-level moisture through dense shrub thickets [25] and serve as high-traffic host corridors, resulting in elevated parasite densities. Grassland systems range from xeric pastures dominated by *Scabiosa columbaria*, which remain high-risk due to constant wildlife presence despite high solar exposure [43], to intensively mown lawns where frequent biomass removal creates unfavorable, low-humidity conditions for ticks. In contrast, irrigated gardens and parks attract rodents and birds, key hosts for immature tick stages, due to their structural heterogeneity and moisture [44]. Finally, secondary succession habitats (“urban jungle”), such as abandoned quarries and ruins, feature dense *Hedera helix* mats that create humid micro-niches for reptiles (*Podarcis sicula*) and small mammals [45]; together with *Smilax aspera* and *Ecballium elaterium*, these sites function as isolated but highly active micro-foci of tick infestation [46,47,48]. Table 1 summarizes the ecological characteristics of the sampling habitats, including dominant vegetation, microclimatic refugia, and anthropogenic influences.

#### 2.1.2. Tick Sampling, Climatic Conditions Assessment and Faunal Similarity

Tick sampling utilized a combination of the standard flagging/dragging method [51] across various habitats using a 1 m^2^ flag and collection from hosts (donkeys and humans) [52]. Sampling was conducted at irregular intervals throughout the study period (15 June 2020–30 November 2022), with field exits performed opportunistically based on site accessibility and weather conditions. Since the primary objective of this study was to survey species diversity and the presence of pathogens rather than standardized seasonal abundance patterns, population phenology was not continuously monitored; however, to ensure methodological consistency, each standardized flagging session was strictly timed at 1 h. Collected ticks were immediately preserved at +4 °C.

Morphological identification of all life stages (adults, nymphs, and larvae) was performed using a stereo microscope Olympus SZX9 (Olympus Corporation, Tokyo, Japan), according to standard taxonomic keys for the Mediterranean fauna [2,53,54]. To document the findings, all specimens were photographed with a digital camera Olympus SC50 (EVIDENT, Münster, Germany). While we acknowledge the absence of molecular confirmation in the present study as a potential limitation, the reliability of our morphological identification is supported by our previous research in the nearby region of Istria [14]. In that study, 100% of the specimens identified morphologically by the same researchers were subsequently confirmed as accurate through DNA barcoding and preserved at −80 °C for molecular analysis. During the sampling process, environmental parameters, including temperature, relative humidity and wind speed—were obtained from the official meteorological station located within Brijuni National Park (DHMZ). Given the relatively small and uniform study area, these data were considered representative for all sampling micro-locations at the specific times of collection. Wind speed was monitored as a potential factor influencing air desiccation, which is of particular relevance given the low-lying island topography of the study sites. The correlation between the number of collected ticks (N) and the climatic conditions recorded during the sampling (air temperature (°C), relative air humidity (%) and the wind speed (km/h), was tested with the Pearson’s correlation coefficient (r, *p* < 0.05) using the Statistica Version 14.1.0.8 TIBCO Software Inc. (Cloud Software Group, Inc. (2023). Data Science Workbench, version 14. http://tibco.com, San Ramon, CA 94583, USA). Classifications of magnitude were interpreted as follows: 0.00 < 0.20 = very weak correlations; 0.20–0.39 = weak correlations; 0.40–0.59 = moderate correlations; 0.60–0.79 = strong correlations; correlations > 0.80 as very strong. Analysis of tick fauna diversity among different vegetation types was conducted using the Sørensen faunal similarity index.

### 2.2. Molecular Pathogen Detection

For DNA extraction, ticks were grouped into pools based on species, developmental stage, and sampling location, as detailed in Table 2. A total of 45 pools were prepared from 245 tick individuals. Due to the scope of a larger regional surveillance project and resource optimization, a representative subsample of 216 randomly selected *I. ricinus* individuals was further processed for pathogen detection. The specific primers and probes used for the qPCR screening of the six zoonotic bacterial pathogens are detailed in Table 3, which illustrates the targeted gene sequences, amplicon lengths, primer and probe names, and their respective sequences (5′–3′) for each analyzed pathogen [55].

Interpretation of the results was based on the cycle threshold (Ct) values and the morphology of the amplification curves. A sample was defined as positive when the Ct value was <35, accompanied by a clear logarithmic increase in fluorescence. The use of specific TaqMan probes ensured high diagnostic specificity, as previously established for this method. To validate the runs, positive genomic DNA of target pathogens and non-template controls (NTC) were included in each qPCR plate. No sequencing of amplicons was performed, as the utilized TaqMan-based assay is specifically designed and validated for the definitive identification of these pathogens without the need for further confirmation [55].

Prior to nucleic acid extraction, ticks were rinsed in 70% ethanol and RNase-free water. The global importance of ticks as vectors for various pathogens necessitates rigorous molecular screening protocols [56,57]. For DNA extraction, ticks were grouped into pools based on species, developmental stage, and sampling location, as detailed in Table 3.

Total DNA was extracted from 45 pools using the NucleoSpin^®^ DNA Insect kit (Macherey-Nagel GmbH & Co. KG, Düren, Germany) according to the manufacturer’s instructions. The concentration and purity of the extracted DNA were verified using a NanoPhotometer P300 (IMPLEN GmbH, Munich, Germany).

Screening for six zoonotic bacterial pathogens (*A. phagocytophilum*, Bbsl., *B. miyamotoi*, *F. tularensis*, *R. conorii*, and *E. canis*) was performed via quantitative Real-Time PCR (qPCR) on a QuantStudio 5 Real-Time PCR System (Applied Biosystems, Foster City, CA, USA) using the highQu 4X 1Step RT qPCR Probe ROX L Kit (highQu, Kraichtal, Germany). The screening followed the validated high-throughput protocol described by Michelet et al. (2014) [55], which is widely recognized for the large-scale surveillance of tick-borne pathogens [56]. Given the status of various pathogens as emerging threats in Europe [57] and the changing climate niches of invasive species like *H. marginatum* [58], such high-throughput methods are essential for accurate diagnostic evaluation [59].

### 2.3. Minimal Infection Rate and Co-Infection Assessment

To estimate the prevalence of pathogens in the collected tick population, the Minimal Infection Rate (MIR) was calculated for each detected pathogen based on the results of the molecular analysis of pooled samples [60]. MIR represents the minimum number of infected ticks per 100 tested specimens, if each positive pool contains only a single infected tick specimen. MIR was calculated using the following Formula (1):

(1) $$MIR=\frac{Number\;of\;Positive\;Pools}{Total\;Number\;of\;Tick\;Tested}\times 100\%$$

The MIR values obtained were expressed as the number of infected ticks per 100 ticks. MIR was used as a conservative indicator of pathogen prevalence, providing a lower bound estimate of the actual infection rate within the vector population.

To assess the relative occurrence of simultaneous infections within the collected ticks, the Co-infection Rate (CR) was calculated [55,61]. The CR is defined as the percentage of all pathogen-positive pools that contain two or more different pathogens.

### 2.4. GIS Mapping

Spatial distribution and visualization of tick collection locations, associated habitats, and the presence of pathogens were performed using the GIS application QGIS, version 3.28 (QGIS Association, Gossau, Zurich, Switzerland). This tool enabled the integration of GPS field data with pathogen detection results to map zoonotic risk across Istria County.

## 3. Results

### 3.1. The Spatio-Temporal Distribution of Collected Ticks

A total of 587 ticks belonging to four genera (*Haemaphysalis*, *Hyalomma*, *Ixodes*, *Rhipicephalus*) were collected during the study period, with *I. ricinus* being the dominant species (Σ =552, larvae (L) = 28; nymphs (N) = 468; females (F) = 37, males (M) = 19). Regarding the developmental stages of the entire collection, the majority were nymphs (N = 475), followed by adults (Σ = 84; M = 34, F = 50) and larvae (L = 28). *Hyalomma marginatum* and *Haemaphysalis punctata* were recorded only occasionally, with Σ = 16 (F = 12; M = 4) and Σ = 13 (N = 2; M = 11) specimens, respectively. Other taxa were rare; the ornithophilic tick *I. frontalis* was represented by just Σ = 5 specimens (N = 4; F = 1), while the *Rhipicephalus sanguineus* was exceptionally scarce, with only one individual (N = 1) identified throughout the entire sampling period.

**Figure 2:**
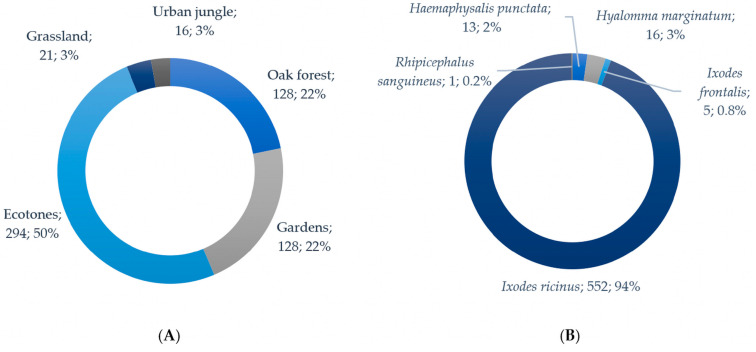
(**A**) total number of tick specimens collected per habitat type in Brijuni National Park; (**B**) taxonomic composition of tick species identified in the study area.

Using the flagging method 574 (97.8%) individuals were sampled during 24 h altogether (4.8 h per sampling location on average). Additionally, 12 (2.0%) tick specimens were collected on a donkey, and one tick (0.2%) was sampled from a human host. The 12 specimens from the donkey were identified as adult *H. marginatum* (F = 8, M = 4), all of which were collected in the grassland systems (pastures). The specimen collected from the human host was identified as an *I. ricinus* nymph; it was analyzed individually and tested negative for all screened pathogens. Figure 2A illustrates the total number of ticks collected across different habitat types, whereas Figure 2B details the taxonomic composition of the identified species. 

The overall number of ticks collected on different habitat types via flagging method varied from 9 (in grassland) to 294 (in ecotones) individuals. Total and relative number (Ixodidae/h) of ixodid ticks collected on different habitat types, depending on the sampling period (h), are shown in Table 4.

**Figure 3:**
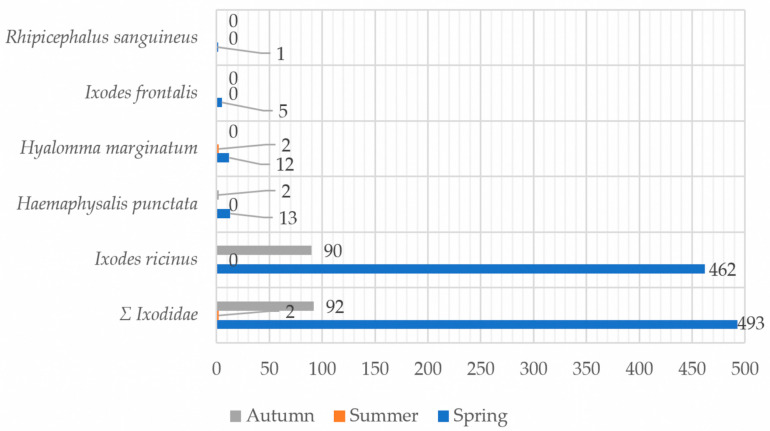
Total number of collected ticks by species across different sampling periods (spring, summer, and autumn) from 2020 to 2022.

The highest number of collected ticks, among all the species, was recorded during spring sampling, with a secondary, minor peak observed in autumn (Figure 3). Sampling was conducted opportunistically at irregular intervals between June 2020 and November 2022; therefore, these results represent the collected sample distribution rather than a standardized assessment of seasonal tick presence.

### 3.2. Correlation of Environmental Factors and Standardized Tick Catch (N/h)

The average annual air temperatures in Croatia from 2020 to 2022 were above the multi-annual average when compared to the 30-year reference climate period (1981–2010: before 2023, 1991–2020: after 2023). According to the associated percentile values, thermal conditions (air temperature anomalies), were very warm in 2020, warm in 2021 and extremely warm in 2022. The annual mean precipitation, on the other hand, varied from normal in 2020 to dry in 2021 and 2022. If we look closer into season anomalies, corresponding average spring temperatures in 2020 were within the category warm, cold in 2021 and normal in 2022. Precipitation amounts in springtime 2020 were described as very dry, then normal in 2021 and again very dry in 2022.

The correlation between climatic parameters (air temperature (°C), relative air humidity (%) and the wind speed (km/h) and number of collected ticks (N) across different habitat types, as determined by a correlation analysis, is illustrated in Table 5.

### 3.3. Faunal Similarity

Based on the Sørenson index (Table 6), the highest faunal similarity (66.7%) was observed between cultivated grasslands & gardens and ecotones (forest edges & shrublands), as well as between grassland systems (pastures) and urban jungles. Conversely, the lowest similarity (40.0%) was found when comparing the climatogenic holm oak forest with both cultivated grasslands & gardens and ecotones. A similarly low index was noted between urban jungles and both cultivated grasslands & gardens and ecotones.

### 3.4. The Spatial Distribution of Determined Tick-Borne Pathogens

The analysis of collected tick pools (Table 7) revealed a significant presence of Bbsl and *A. phagocytophilum*, primarily associated with *I. ricinus*. Both pathogens demonstrated a broad ecological distribution; Bbsl was identified in 11 pools across various habitats, while *A. phagocytophilum* was found in 8 pools. The highest prevalence for both was recorded in ecotones and climatogenic Holm oak forests, where they were detected in 5 separate pools each. Notably, while *A. phagocytophilum* was predominantly found in *I. ricinus*, it was also identified in one pool of *H. marginatum* within grassland systems. *I. ricinus* additionally served as the vector for *E. canis*, identified in 3 pools across all surveyed environments, including anthropogenic sites like cultivated gardens. Furthermore, *I. frontalis* was confirmed as a carrier for *B. divergens* (1 pool) and Bbsl (1 pool) in forest and ecotone settings. In contrast, *F. tularensis* and *B. myamotoi* were not detected (0 pools) in any of the analyzed samples.

**Figure 4:**
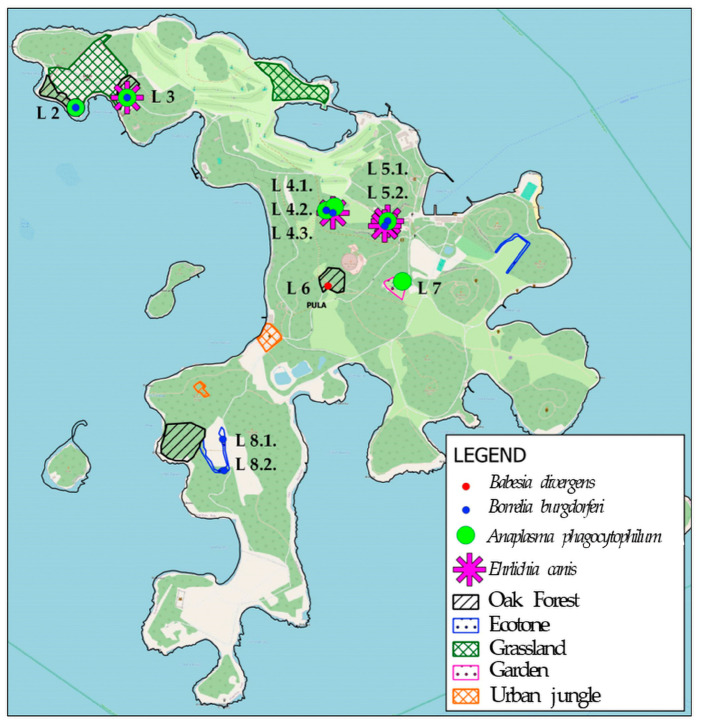
Veliki Brijuni—geographical mapping of *Babesia divergens*, *Borrelia burgdorferi*, *Anaplasma phagocytophilum* and *Ehrlichia canis* detected in collected ticks. Labels L 2 through L 8.2 represent the specific sampling locations (micro-localities) within the National Park across different habitat types (oak forest, ecotones, and gardens) where the pathogens were identified.

The spatial distribution of tick-borne pathogens across the study area on Veliki Brijuni, with sampling sites marked by pathogen-specific symbols to denote detected species is illustrated in Figure 4.

**Figure 5:**
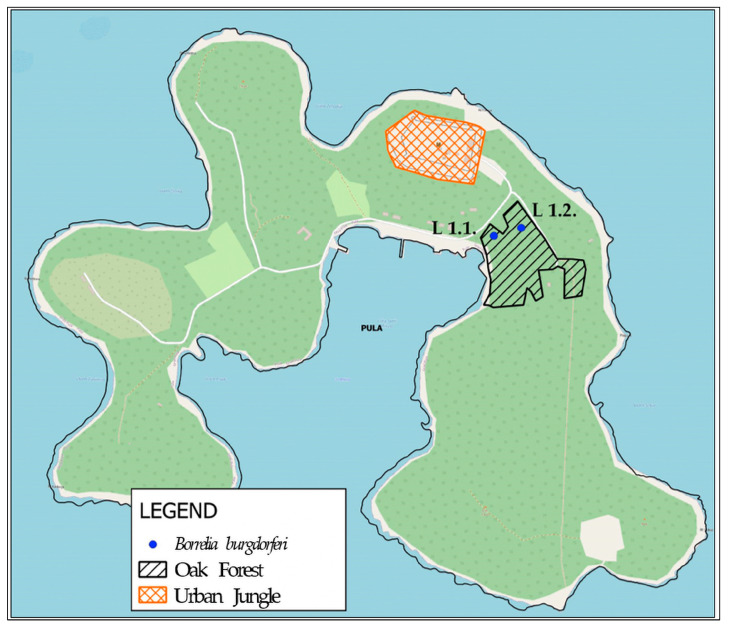
Spatial distribution of investigated locations and *Borrelia burgdorferi* pathogen detection on Mali Brijun. Labels L 1.1. and L 1.2. represent the specific sampling locations (micro-localities) within the National Park across different habitat types (oak forest, and urban jungle) where the pathogens were identified.

On Mali Brijun Island (Figure 5), ticks were detected at two out of the four investigated locations (50%). Only the species *I. ricinus* were collected at both positive sites. Molecular analysis of the collected ticks from Mali Brijun confirmed the active circulation of the pathogen Bbsl. Both positive locations were situated within the holm oak forest habitat.

Habitat-specific distribution and pathogen co-infection patterns are summarized in Table 8. Heterogeneous spatial distribution of tick-borne pathogens was determined in *Ixodes ricinus* specimens, characterized by a high frequency of co-infections at several microlocations (e.g., location L3 (holm oak forest) and location L5.2 (garden), where three pathogens—Bbsl, *A. phagocytophilum*, and *E. canis*—were simultaneously detected.

### 3.5. Minimal Infection Rate (MIR) and Co-Infection Rate (CR)

Molecular analysis of pooled samples confirmed the presence of three pathogens—Bbsl, *A. phagocytophilum*, *E. canis*, while three pathogens were not detected, *R. conorii*, *B. myamotoi*, and *F. tularensis*, in any tested tick pools. Bbsl exhibited the highest Minimal Infection Rate (MIR = 3.05), followed by *A. phagocytophilum* (MIR = 1.81), and *E. canis* (MIR = 1.58). These findings establish LB as the most significant vector-borne risk within the study area.

Molecular analysis confirmed active pathogen co-circulation at the habitat level. Out of 17 tested tick pools, five were positive for the simultaneous presence of two or three pathogens. The Co-infection Rate (CR) was 29.41%. Co-circulation was observed as both triple Co-circulation (involving *E. canis*, *A. phagocytophilum*, and Bbsl in two pools) and double Co-circulation (primarily combinations of Bbsl with *E. canis* or *A. phagocytophilum* in three pools).

## 4. Discussion

The dominance of the species *I. ricinus*, accounting for 94.0% of the total sample in our study confirms the status of Brijuni National Park as a stable and high-risk habitat for the primary vector of zoonotic pathogens in this part of Europe [22]. Such a high prevalence of a single species in a mixed Mediterranean habitat indicates the exceptional adaptability of these ticks to the island’s specific microclimatic conditions.

The recorded bimodal activity pattern, characterized by a pronounced peak in spring and a complete absence of ticks during the summer months, represents a classic example of “summer diapause” triggered by extreme temperatures and low air humidity [22]. Ticks retreat into deeper layers of leaf litter to avoid lethal desiccation, which is of critical importance for understanding seasonal risk: the greatest danger to visitors is not actually during the peak tourist season in August, but rather during the more humid pre-season period [5,30,43].

The stability of these populations is inseparable from the presence of a dense population of non-native wildlife. Fallow deer, axis deer, and mouflon, which inhabit Brijuni in large numbers within a relatively small area, act as key “hosts” for the adult stages of ticks [22,23,30,37]. Their free movement across the entire island, including the immediate vicinity of human settlements and tourist zones, enables the continuous dispersal of ticks and maintains the high infectious potential of the environment.

Spatial analysis conducted via GIS revealed that ecotones, the transitional zones between forests and open grasslands, represent the primary epidemiological foci. More than 50% of all samples were collected in these zones, confirming the so-called “edge effect” [22,62,63]. This phenomenon is the result of ecological synergy: dense maquis and garrigue vegetation provide the necessary moisture and protection from the sun, while open paths and meadows serve as corridors for host movement and grazing [25,32,62]. In these transition zones, a significant positive relationship was observed between air temperature and the number of active ticks, suggesting that moderate temperature increases within these stable environments promote questing behavior. Conversely, wind speed acted as a significant limiting factor in ecotones, likely due to its effect on increasing the risk of desiccation, which forces ticks to retreat to lower, more humid vegetation layers. Our findings showed that significant correlations with temperature and wind speed occurred only in ecotones can be explained by the high sensitivity of these transitional zones to environmental fluctuations. Unlike stable forest interiors, ecotones lack a dense canopy buffer, making ticks more responsive to immediate meteorological changes [13,58]. While moderate warming promotes questing activity in these areas, higher wind speeds likely increase the desiccation risk, forcing ticks to seek refuge in lower vegetation layers to maintain water balance, a dynamic particularly relevant in fragmented habitats [13,51].

The results from cultivated gardens and parks are particularly intriguing. Although it might intuitively be expected that landscaped areas would be safer, our research shows a tick density that is entirely comparable to that found in wild, indigenous holm oak forests. The primary reason is likely the human factor, i.e., continuous irrigation during summer months creates artificial microclimatic refugia [22,45]. While the surrounding nature experiences drought that reduces tick activity, irrigated lawns maintain the humidity levels critical for their survival. This creates a significant trap for tourists who feel safe in the landscaped parts of the park, unaware that these zones, due to moisture and the proximity of animals, are high-risk areas [44,64].

Comparison of hard tick fauna in different habitat types via the Sørenson index showed the lowest similarity between the oak forest and gardens, while the fauna of gardens and ecotones, as well as grassland and urban jungle, exhibited the highest similarity to each other.

The detection of three pathogens (Bbsl, *A. phagocytophilum*, and *E. canis*) confirms that Brijuni NP functions as a hyperendemic focus [22,55]. Our findings are consistent with current models of tick-borne pathogen distribution and ecological niche modeling [65]. Seasonal variations in tick activity, observed in other parts of Europe [66,67], further emphasize the risk of rickettsioses and other emerging infections [68]. The high prevalence of Bbsl reflects the increasing incidence of LB reported in systematic reviews across the continent [69,70]. Consequently, the highest infection rate (MIR = 3.05) recorded for Bbsl places LB at the center of the island’s public health strategy [22,71]. The study highlights a substantial co-infection Rate (CR = 29.41%), which warrants further attention.

Although these data were technically derived from the analysis of pooled samples, they serve as a powerful indicator of actual epidemiological risk in the field [60,61]. This aligns with broader European trends indicating an increase in the complexity of pathogen transmission cycles [62]. Such a high frequency of multiple pathogens within the same samples points to the intensive circulation of various agents within the same microlocations and shared infection reservoirs, primarily wildlife [40,48]. From a clinical perspective, this suggests the probability of a tick bite simultaneously transmitting a “cocktail” of pathogens, which can lead to complex synergistic infections [61,72,73]. It is well-documented that co-infection with *A. phagocytophilum* and Bbsl often leads to more severe and complicated forms of the disease [72,74,75]. *Anaplasma* attacks white blood cells and causes temporary immunosuppression, allowing *Borrelia* to spread more rapidly and aggressively through host tissues [72,75,76]. Furthermore, recent studies emphasize that such co-exposures can significantly alter the hematological profile of the host, further complicating the clinical assessment [77]. For physicians, this means that patients from Brijuni may present with atypical symptoms that do not correspond to the classic erythema migrans, complicating diagnosis and requiring a broader spectrum of antibiotic therapy [74,76].

The absence of the pathogen *R. conorii*, the causative agent of Mediterranean spotted fever, is likely the result of the specific regime in Brijuni NP where the movement of domestic dogs, the primary hosts for the tick *R. sanguineus*, is strictly controlled [1,2]. This aligns with broader epidemiological observations in the Mediterranean region, where the distribution of *Rickettsia* species is closely linked to host availability and environmental factors [78,79]. Consequently, this indicates that zoonotic cycles in the park primarily occur between wild animals, birds, and small rodents [40,43].

On the other hand, the detection of the species *H. marginatum* (3.0%), although in small numbers, warrants vigilance. As the primary vector of the CCHF virus, this tick prefers warmer and drier habitats [18,58]. Its presence on Brijuni suggests that ecological niches in the northern Adriatic are changing under the influence of climate change, favoring species that were previously characteristic only of more southern regions [8,17]. Furthermore, the role of birds in this system cannot be ignored; the detection of pathogens in the bird-specific species *I. frontalis* confirms that migratory species serve as “air bridges” for the importation of new pathogen strains from distant areas [28,50].

This research proves that island isolation does not provide protection against vector-borne diseases; on the contrary, under the specific conditions of Brijuni NP, it creates an intensive and closed system of pathogen circulation. The use of GIS modeling has allowed us to precisely identify risk “hotspots” for the first time, providing a foundation for a modern approach to health protection. The application of the “One Health” concept on Brijuni is no longer an option but a necessity [11,21,80].

Given the high tick density in zones with high human frequency, park management must integrate targeted environmental maintenance, such as regular mowing of path edges and thinning of maquis near tourist routes to reduce the humidity levels ticks require [63,64]. Furthermore, controlling wildlife populations within ecological limits is crucial for breaking the tick reproductive cycle [23,40]. Finally, precise visitor education, informing them of the specific risks in irrigated gardens and at forest edges rather than providing general warnings, represents the most effective way to reduce the incidence of tick-borne diseases in this protected area [5,22].

Several limitations of the present study should be acknowledged. First, the identification of ticks was based solely on morphological characteristics using standard taxonomic keys. While these keys are the gold standard for the Mediterranean fauna, we recognize that the absence of molecular confirmation for all specimens remains a potential constraint, particularly for immature stages. Second, due to the large number of samples, testing was performed in pools rather than individually. Consequently, the results are expressed as the Minimum Infection Rate (MIR), which provides a conservative estimate. This approach may underestimate the true pathogen prevalence, as it assumes only one infected individual per positive pool. These factors should be considered when interpreting the absolute infection frequencies within the study area.

## 5. Conclusions

The investigation into the ecological determinants and spatial distribution of ticks within Brijuni National Park provides essential insights into the dynamics of tick-borne pathogens in this unique island ecosystem. The absolute dominance of *I. ricinus* confirms its role as the primary vector in the area, exhibiting a seasonal activity pattern that is finely tuned to the Mediterranean microclimate. This stable population is fundamentally sustained by the high density of non-native wildlife, which serves as the primary reservoir and host, ensuring the continuity of the tick life cycle across the islands. In contrast, the absence of *R. conorii* and very low recorded abundance of its’ vector, *R. sanguineus*, suggests that the control of domestic animals within the park successfully prevents certain zoonotic cycles from establishing.

Spatial analysis has been instrumental in identifying critical habitats, demonstrating that ecotones represent the highest risk zones due to their specific microclimatic conditions. A particularly significant finding is the high tick density within cultivated gardens and parks, suggesting that anthropogenic influences—specifically irrigation—create artificial refugia. These managed areas sustain tick activity even during dry periods, thereby increasing the potential exposure for visitors in locations typically perceived as safe.

Furthermore, the molecular evidence confirms that the park functions as a hyperendemic focus for the co-circulation of several pathogens, with LB emerging as the most prominent public health threat. The high frequency of multiple pathogens within the same samples indicates a significant probability of simultaneous transmission, presenting a complex challenge for clinical practice. Such findings necessitate increased vigilance, as the synergistic effects of these pathogens can lead to atypical clinical presentations and complicate standard diagnostic procedures.

The detection of the thermophilic species *H. marginatum*, alongside pathogens found in bird-specific ticks, further warns of shifting ecological niches influenced by climate change and the role of migratory birds in introducing new disease agents to the northern Adriatic. Ultimately, these results mandate the implementation of a “One Health” approach to park management. By focusing on targeted vegetation maintenance in identified hotspots, managing wildlife populations, and providing precise education to tourists, the park can ensure a safer coexistence between people and the natural environment in this protected area.

## Figures and Tables

**Table 1:** Summary of ecological conditions by habitat type in Brijuni NP.

Habitat Type	Dominant Vegetation/Taxa	Microclimate	Anthropogenic /Biotic Influence
oak forest (climatogenic holm oak forest comunity)	*Quercus ilex*, *Viburnum tinus*, *Ruscus aculeatus*	High humidity (78–85% in litter), stable thermal refugia [49]	Low disturbance, high wildlife density (fallow deer, mouflon)
ecotones (forest edges & shrublands)	*Pistacia lentiscus*, *Pistacia terebinthus*, *Myrtus communis*	Moisture retention near soil surface due to dense thickets [48]	Maximum wildlife frequency; high-risk interface for vector exposure [11,50]
grassland (pastures)	*Scabiosa columbaria*, Xeric grasses	Dry/High solar radiation [49]	Intensive grazing regime; sustained tick population via blood meals
gardens (cultivated grasslands and gardens)	Mown graminoids, ornamental shrubs	Variable; irrigation maintains high ground-level humidity [45]	Intensive mechanical maintenance (mowing) vs. high rodent/bird activity
urban jungle (successional)	*Hedera helix*, *Smilax aspera*, *Ecballium elaterium*	Xerothermic but with protected, humid micro-niches	Secondary succession [50]; focus for reptiles (*Podarcis sicula*) & rodents [46,47,48]

**Table 2:** Number of tick individuals and pools prepared for qPCR testing.

Tick Species	Total No. of Individuals (n)	Number of Pools	Pool Size (Range) *
*Ixodes ricinus*	216	33	1–40
*Haemaphysalis punctata*	13	8	1–5
*Hyalomma marginatum*	12	2	1–7
*Ixodes frontalis*	3	1	3
*Rhipicephalus sanguineus*	1	1	1

* pools contained mixed developmental stages (nymphs and adults).

**Table 3:** List of pathogens, targeted genes and primers (F—Forward, R—Reverse, P—Probe).

Pathogen Species	Target Gene	Length (bp)	Primer Name	Primer Sequence (5′–3′)	Reference
*Borrelia* *burgdorferi* s.l.	23S rRNA	73	Bo_bu_sl_23S_F	GAGTCTTAAAAGGGCGATTTAGT	[55]
			Bo_bu_sl_23S_R	CTTCAGCCTGGCCATAAATAG	
			Bo_bu_sl_23S_P	AGATGTGGTAGACCCGAAGCCGAGT	
*Borrelia* *miyamotoi*	glpQ	93	BmglpQ_F	CCGTTGGAATTACTTTGTATGTT	[55]
			BmglpQ_R	TTCCAGGTATTGCAGTCTCAG	
			BmglpQ_P	AGAAACGTTCAGGGCCAGTTTACCAG	
*Rickettsia* *conorii*	23S-5S ITS	118	Ri_co_ITS_F	CTCACAAAGTTATCAGGTTAAATAG	[55]
			Ri_co_ITS_R	CGATACTCAGCAAAATAATTCTCG	
			Ri_co_ITS_P	CTGGATATCGTGGCAGGGCTACAGTAT	
*Francisella* *tularensis*	tul4	76	Fr_tu_tul4_F	ACCCACAAGGAAGTGTAAGATTA	[55]
			Fr_tu_tul4_R	GTAATTGGGAAGCTTGTATCATG	
			Fr_tu_tul4_P	AATGGCAGGCTCCAGAAGGTTCTAAGT	
*Anaplasma* *phagocytophilum*	msp2	77	An_ph_msp2_F	GCTATGGAAGGCAGTGTTGG	[55]
			An_ph_msp2_R	GTCTTGAAGCGCTCGTAACC	
			An_ph_msp2_P	AATCTCAAGCTCAACCCTGGCACCAC	
*Ehrlichia* *canis*	dsb	110	Eh_ca_dsb_F	AATACTTGGTGAGTCTTCACTCA	[55]
			Eh_ca_dsb_R	GTTGCTTGTAATGTAGTGCTGC	
			Eh_ca_dsb_P	AAGTTGCCCAAGCAGCACTAGCTGTAC	

**Table 4:** Species composition, total count (n), and efficiency (ticks/h) of tick flagging across different habitat types for *Ixodes ricinus* speciemens.

Habitat Type	Sampling Duration (h)	Σ *Ixodes ricinus*
		*Ixodes ricinus*/h
oak forest	5	126
		25.2
gardens	7	118
		16.9
ecotones	7	284
		40.6
grassland	2	9
		4.5
urban jungle	3	14
		4.7
Σ	24	551

**Table 5:** Correlation between climatic parameters and the number of ixodid ticks (N) sampled by flagging method at different habitat types and the whole Brijuni National Park (BNP).

Climatic Parameters	Oak Forest		Gardens		Ecotones		BNP	
	N		N		N		N	
	*r*	*p*	*r*	*p*	*r*	*p*	*r*	*p*
Air temperature	0.4863	0.5575	−0.5258	0.1302	0.8137	0.0305	−0.0205	0.4770
Relative air humidity	0.3591	0.8685	−0.5957	0.1254	0.3798	0.7384	0.1680	0.9990
Wind speed	−0.3731	0.2573	0.5106	0.7573	−0.7301	0.0353	−0.1822	0.0629

**Table 6:** Sørenson index values for hard tick faunas in different habitat types at BNP.

Habitat Type	Oak Forest	Gardens	Ecotones	Grassland
oak forest	-	-	-	-
gardens	40	-	-	-
ecotones	40	66.7	-	-
grassland	50	50	50	-
urban jungle	50	40	40	66.7

**Table 7:** Distribution of pathogens across tick species and habitat types.

Pathogen	Species	N Pool	Habitat Type
*Francisella tularensis*	0	0	0
*B. myamotoi*	0	0	0
*Babesia divergens*	*Ixodes frontalis*	1	climatogenic holm oak forest
*Borrelia burgdorferi s.l.*	*Ixodes frontalis*	1	ecotones (forest edges & shrublands)
*Borrelia burgdorferi s.l.*	*Ixodes ricinus*	4	ecotones (forest edges & shrublands)
*Borrelia burgdorferi s.l.*	*Ixodes ricinus*	5	climatogenic holm oak forest
*Borrelia burgdorferi s.l.*	*Ixodes ricinus*	1	cultivated grasslands & gardens
*Anaplasma phagocytophilum*	*Ixodes ricinus*	5	ecotones (forest edges & shrublands)
*Anaplasma phagocytophilum*	*Hyalomma marginatum*	1	grassland systems (pastures)
*Anaplasma phagocytophilum*	*Ixodes ricinus*	1	climatogenic holm oak forest
*Anaplasma phagocytophilum*	*Ixodes ricinus*	1	cultivated grasslands & gardens
*Ehrlichia canis*	*Ixodes ricinus*	1	cultivated grasslands & gardens
*Ehrlichia canis*	*Ixodes ricinus*	1	climatogenic holm oak forest
*Ehrlichia canis*	*Ixodes ricinus*	1	ecotones (forest edges & shrublands)

**Table 8:** Overview of sampling locations, habitat types and pathogens identified within *Ixodes ricinus* specimens.

Location	Habitat Type	Tick Species	Pathogen
L 1.1.	oak forest	*Ixodes ricinus*	*Borrelia burgdorferi*
L 1.2.	oak forest	*Ixodes ricinus*	*Borrelia burgdorferi*
L 2	oak forest	*Ixodes ricinus*	*Borrelia burgdorferi* *Anaplasma phagocytophilum*
L 3	oak forest	*Ixodes ricinus*	*Borrelia burgdorferi* *Anaplasma phagocytophilum* *Ehrlichia canis*
L 4.1.	ecoton	*Ixodes ricinus*	*Borrelia burgdorferi* *Anaplasma phagocytophilum*
L 4.2.	ecoton	*Ixodes ricinus*	*Anaplasma phagocytophilum*
L 4.3.	ecoton	*Ixodes ricinus*	*Borrelia burgdorferi* *Ehrlichia canis*
L 5.1.	garden	*Ixodes ricinus*	*Borrelia burgdorferi* *Ehrlichia canis*
L 5.2.	garden	*Ixodes ricinus*	*Borrelia burgdorferi* *Anaplasma phagocytophilum* *Ehrlichia canis*
L 6	oak forest	*Ixodes ricinus*	*Babesia divergens*
L 7	garden	*Ixodes ricinus*	*Anaplasma phagocytophilum*
L 8.1.	ecoton	*Ixodes ricinus*	*Borrelia burgdorferi*
L 8.2.	ecoton	*Ixodes ricinus*	*Borrelia burgdorferi*

## Data Availability

Raw data were generated at the Teaching Institute of Public Health of Istria County. Derived data supporting the findings of this study are available from the corresponding author on request. We agree with the full transparency of the data if needed. For raw data related to this article, please contact M.C. at maja.cvek@zzjziz.hr.
